# Short-time repeat high-risk HPV testing by self-sampling for screening of cervical cancer

**DOI:** 10.1038/bjc.2011.277

**Published:** 2011-08-02

**Authors:** U Gyllensten, K Sanner, I Gustavsson, M Lindell, I Wikström, E Wilander

**Affiliations:** 1 Department of Immunology, Genetics and Pathology, Rudbeck Laboratory, SciLifeLab, Uppsala University Hospital, Uppsala, Sweden; 2 Department of Women's and Children's Health, Uppsala University, Uppsala University Hospital, Uppsala, Sweden

**Keywords:** cervix, screening, carcinoma, HPV test, persistence, self-sampling

## Abstract

**Background:**

Testing for high-risk human papillomavirus (HPV) in primary screening for cervical cancer is considered more sensitive, but less specific, in comparison with Pap-smear cytology. Women with persistent HPV infections have a higher risk of developing cervical intraepithelial neoplasia 2+ (CIN2+) lesions. This study was performed to evaluate the gain in specificity for detection of histologically confirmed CIN2+ lesions achieved by short-time repeat testing for high-risk HPV in women aged 30–65 years, with the primary sample for HPV analysis taken by self-sampling.

**Methods:**

A total of 8000 women in Uppsala County, aged 30–65 years, who had not attended organised screening for 6 years or longer, were offered self-sampling of vaginal fluid at home and the samples sent for HPV typing. Of these, 8% (669) were not possible to contact or had performed hysterectomy. Women positive for high-risk HPV in the self-sampling test were invited for a follow-up HPV test and a cervical biopsy on average 3 months after the initial HPV test.

**Results:**

In all, 39% (2850/7331) of invited women chose to perform self-sampling of vaginal fluid at home. High-risk HPV infection was found in 6.6% (188) of the women. In all, 89% of the women testing HPV positive performed a follow-up examination, on average 2.7 months, after the first test and 59% of these women were HPV positive in the follow-up test. The prevalence of CIN2+ lesions in women with an initial HPV-positive test was 23% (95% CI 18–30%) and in women with two consecutive HPV-positive tests was 41% (95% CI 31–51%). In women with two positive HPV tests, the prevalence of CIN2+ lesions varied from 49% in women at age 30–39 years to 24% in women at age 50–65 years. Short-time repeat HPV testing increased the specificity for detection of CIN2+ lesions from about 94.2% to 97.8%. The most prevalent HPV types were HPV16 (32%), followed by HPV18/45 (19%) and HPV 33/52/58 (19%).

**Conclusion:**

The short-time persistence of high-risk HPV infection in this age group was about 60%. Repeat testing for high-risk HPV using self-sampling of vaginal fluid can be used to increase the specificity in the screening for cervical cancer in women aged 30–65 years.

The introduction of organised cytological screening in Sweden in the late 1960s has resulted in a decrease of the prevalence of cervical cancer by ∼50% (Bergstrom *et al*, 1999). However, not all women have attend the organised screening programme and a large fraction of cervical cancers occur in women who have not participated in the cytological screening (Andrae *et al*, 2008). By offering self-sampling at home, the population coverage of the screening can be improved (Sanner *et al*, 2009; Gok *et al*, 2010).

Infection with high-risk types of human papillomavirus (HPV) is the dominant risk factor for development of cervical carcinoma (zur Hausen, 1991), and it has been shown that testing for high-risk HPV infection can be used as an adjunct test or an alternative to Pap-smear screening (Wright *et al*, 2004; Cuschieri and Cubie, 2005; Meijer *et al*, 2009). Testing for high-risk HPV in primary screening has also been shown to be twice as sensitive as Pap-smear screening for detection of premalignant (cervical intraepithelial neoplasia 2+ CIN2+) cervical lesions (Ronco *et al*, 2010). In postmenopausal women, the difference in sensitivity between cytology and HPV typing may be even larger, with testing for high-risk HPV being three times more sensitive than cytological screening for detecting women at risk for tumour development (Gyllensten *et al*, 2010).

The use of testing for high-risk HPV in primary screening is complicated by the low specificity of a single HPV-positive test as an indictor of cervical cancer risk. Many women have transient infections, and HPV testing in primary screening could therefore result in inconvenience if no premalignant cell alterations are diagnosed in the cytological smear and no treatment is available for HPV infection. The value of HPV testing can be increased by triaging with cytology or using p16 staining. However, women with a persistent HPV infection have been shown to be at higher risk of developing cervical cancer (Koshiol *et al*, 2008), indicating that follow-up by repeat typing of HPV could be used to identify women at risk.

Previous studies have evaluated the use of repeated HPV testing when the samples were taken in a clinical setting. In this study, we have evaluated the use of short-time repeat testing for high-risk HPV to identify histological CIN2+ lesions on the cervix, when the first sample for HPV analysis is obtained by self-sampling at home. If short-time repeat testing in combination with self-sampling can be shown to efficiently improve the specificity of testing positive for high-risk HPV, only women with two consecutive positive HPV tests need to be admitted to gynaecological surgery for follow-up and examined by colposcopy for the presence of histological CIN2+ lesions on the cervix.

## Material and methods

During 2008–2010, a total of 8000 women (3000 in 2008 and 2500 per year during 2009–2010) from Uppsala County, between the ages of 30–65 years and who had not attended organised cytological screening for 6 years or longer according to the data registry of the organised screening, were offered self-sampling of vaginal fluid at home using the Qvintip device (Aprovix AB, Uppsala, Sweden; Stenvall *et al*, 2006; Sanner *et al*, 2009). Among the 8000 women invited, 669 were not possible to reach because of inaccurate address information or had a previous operation with hysterectomy.

Of the remaining women (*n*=7331), 2850 (39%) chose to participate and sent a vaginal fluid sample to Aprovix for high-risk HPV analysis. The samples were analysed using the hybrid capture II (HC2) method, which detects the presence of any of 13 high-risk HPV types (16, 18, 31, 33, 35, 39, 45, 51, 52, 56, 58, 59 and 68). The result of the HPV analyses was communicated to the participating women within 2–3 weeks after the arrival of the vaginal sample to the laboratory. Women testing positive for HPV were offered an attendance to a gynaecological surgery for a follow-up examination within 1–3 months. At the follow-up visit, the physician performed a colposcopy, collected biopsy samples from the portio (directed biopsy samples or four biopsy samples from the transformation zone if the portio appeared normal) and sampled cervical fluid for a repeat HPV analysis. The second HPV test was performed using the hpVIR method, which detects the following high-risk HPV types 16, 18, 31, 33, 35, 39, 45, 51, 52, 56, 58 and 59 (18 and 45 are detected together, and 33, 52 and 58 as one group; Gustavsson *et al*, 2009a, 2009b). Women with two consecutive positive HPV tests, and either abnormal cytology (ASCUS-CIN3) and/or CIN1–3 at the histopathological analysis, were recommended a further follow-up, or in the case of CIN2+ in the biopsy sample or Pap smear, were treated with surgical cone resection. The cervical biopsy samples were examined by specialists in surgical pathology. When the woman was >30 years old, diagnostic conisation was performed also for women with CIN 1 histopathology in cases when the transformation zone not was visualised. The end point of the study was identification of a CIN2+ in the cervical biopsy or cone resection. The CIN2+ corresponds to the high-grade squamous intraepithelial lesion (HSIL) according to the Bethesda definition of premalignant cell alterations on the cervix.

## Results

A total of 39% (2850/7331) of participating women performed self-sampling of vaginal fluid at home. No difference in attendance rate was seen for different age groups (data not shown). In all, 6.6% (188/2850) of the participants were positive for high-risk HPV in the self-sample. The prevalence of high-risk HPV decreased with age, from 11.5% in women aged 30–39 years to 4.7% in women aged 50–65 years (Table 1). The CIN2+ occurred in 44 of the HPV-positive women (23%, 95% CI 18–30%). The prevalence of CIN2+ among HPV-positive women was 33% in women aged 30–39 years and 14% in women aged 50–65 years (Table 1).

Of the 188 women that were positive for high-risk HPV in the self-sample, 168 performed a follow-up examination, giving a compliance of 89% (168/188). Eleven of the women chose to visit a midwife reception for sampling of cervical smear for cytological screening and HPV analysis. These women are not further described. In three additional women, cervical biopsy samples were collected but no sample for HPV analysis was obtained, and these were also excluded from further analyses.

In the remaining 154 women, cervical biopsies were performed in combination with sampling for a repeat HPV testing at a gynaecological surgery. Five HPV tests were regarded as invalid because of low amount of cells in the sample. In the remaining 149 women with two valid consecutive high-risk HPV tests, of which 88 were positive and 61 negative, the presence of CIN2+ lesions could be evaluated. The mean time between the first and second HPV test was 2.7 months, ranging from 4.0 months for women in the age group of 30–39 years to 1.9 months for women in the age group of 50–65 years.

Repeated HPV positivity was found in 67% of women of age 30–39 years, in 64% in women of age 40–49 years and in 47% of women of age 50–65 years (Table 2). The difference between age groups was not statistically significant (*P*=0.350). Among the 88 women with a persistent HPV infection, 36 (41%, 95% CI 31–51) had histologically defined CIN2+ lesions (Table 2). The prevalence of CIN2+ in women with a short-time persistent HPV infection varied from 49% in women of age 30–39 years to 24% in women of age 50–65 years (Table 2). Among women that were HPV positive in their first test, 23% had CIN2+, whereas 41% of women with two consecutive positive HPV tests had a CIN2+.

Information on the infecting HPV type was obtained for 88 women participating in the second HPV test. HPV16 was the most prevalent type (32%), followed by HPV18/45 (19%) and the group HPV33/52/58 (19%). Among other types, HPV31 occurred in 9%, HPV35 in 3%, HPV 39 in 3%, HPV51 in 3%, HPV6 in 4% and HPV59 in 10% of women positive in the second HPV test. The prevalence of HPV16 decreased with age, from 34% in premenopausal women to 23% in postmenopausal (⩾50 years old) women (*P*<0.02). The specificity of using a single HPV test in screening in comparison with two consecutive HPV tests for detection of histologically defined CIN2+ is shown in Table 3. A single high-risk HPV test in primary screening showed a specificity of 94.2%, whereas applying a repeat high-risk HPV test increased the specificity to 97.8%. The specificity of both primary and repeat high-risk HPV testing increased with age.

## Discussion

In Uppsala County, the coverage in the organised cytological screening is <70% and low in comparison with other counties in Sweden. This was one of the main reasons for introducing self-sampling of vaginal fluid at home in combination with HPV testing as an adjunct in the screening for cervical cancer in 2007 (Sanner *et al*, 2009). The low coverage in the Uppsala County is one possible explanation for the relatively high acceptance rate for self-sampling of vaginal fluid at home (39%) in comparison with other similar studies (Gok *et al*, 2010).

The organised screening in Sweden has reduced the prevalence of cervical cancer from about 1000 cases per year to around 450 cases per year (Bergstrom *et al*, 1999). In a recent nationwide audit in Sweden, it was reported that 65% of women with cervical cancer have not participated in the screening, whereas around 25% of the cervical carcinomas develop among women who regularly have attended the screening and have been repeatedly informed that their smear is normal (Andrae *et al*, 2008). This observation emphasises the need to develop new means of increasing the participation rate and obtain novel screening methods with higher sensitivity than Pap-smear cytology (Sanner *et al*, 2009; Gok *et al*, 2010).

Testing for high-risk HPV in primary screening results in a higher sensitivity in comparison with Pap-smear screening (Wright *et al*, 2004; Cuschieri and Cubie, 2005; Meijer *et al*, 2009). HPV testing identifies around twice as many CIN2+ lesions per 1000 examinations as the regular Pap-smear screening (Ronco *et al*, 2010). The difference between HPV typing and cytology seems to increase with age (Gyllensten *et al*, 2010). An introduction of HPV testing in primary screening is facilitated by its high sensitivity (Wright *et al*, 2004; Cuschieri and Cubie, 2005; Meijer *et al*, 2009) and by its high negative predictive value, indicating that screening intervals can be prolonged (Naucler *et al*, 2007; Kitchener *et al*, 2009), but it has the disadvantage of a low specificity (Ronco *et al*, 2010). A number of studies have shown that repeat testing for HPV has a higher specificity than a single test in primary screening (Plummer *et al*, 2007; Shiffman and Smith, 2007; Rodriguez *et al*, 2008; Castle *et al*, 2009). Further, women with aberrant cytology have a lower clearance rate of high-risk HPV in comparison with women showing normal cytology (Bulkmans *et al*, 2007). A meta-analysis showed that persistent HPV infection is consistently associated with higher risk of CIN2+/HSIL (Koshiol *et al*, 2008).

By repeating the HPV test within a few months of the primary test, and performing clinical follow-up only on women with a short-time persistent HPV infection, we obtained a higher specificity for the entire age spectrum of women 30–65 years of age in comparison with primary high-risk HPV testing. The time interval between the initial and repeat HPV test was chosen to be similar to that used in the follow-up of women with ASCUS and CIN1 alterations in the routine cytological screening. As transient HPV infections are known to remain for 6–24 months, some of the women testing positive in the two subsequent HPV tests are likely to have cleared the virus later. Thus, our study design will underestimated the specificity of repeat HPV testing. However, as information of a high-risk HPV infection may cause discomfort to the women, a reduced length of the time period between samplings was chosen.

We note that there is a lower prevalence of CIN2+ in women with short-time persistent HPV infections in the higher age group. This could be due to the shorter follow-up time in the age group of 50–65 years (1.9 months) relative to the women aged 30–39 years (4 months). Another explanation is that the transformation zone between squamous and glandular mucosa moves upwards in the cervical canal after menopause and it is possible that a number of CIN2+ lesions are not detected in older women despite gynaecological examination, colposcopy and cervical biopsy.

We used two different high-risk HPV tests in the present investigation. Although the hpVIR test is favoured by its ability to be type specific, the self-sampling device Qvintip has been validated in combination with the FDA-approved HC2 method (Stenvall *et al*, 2007; Sanner *et al*, 2009). The two methods give similar but not absolutely identical results with regard to the identification of smears harbouring high-risk HPV (Gustavsson *et al*, 2009a). The hpVIR assay does not include HPV 68. The lack of HPV68 in the hpVIR assay could result in women classified as having a transient rather than persistent infection. This would reduce the apparent value of repeat typing and lead to an underestimation of the specificity. Given the relatively low frequency of this HPV type, the effect is likely to be rather small. Studies are in progress to introduce hpVIR in combination with self-sampling of vaginal fluid at home (Gustavsson *et al*, 2009b).

As expected, HPV 16 and 18/45 were the most common virus types present, together found in 51% of the HPV-positive samples, followed by the group HPV33/52/58 (19%). The prevalence of HPV16 decreased with age from 34% in premenopausal women to 23% in postmenopausal women. This observation may have some significance with respect to the long-term effect of human HPV vaccination, in particular for countries with an organised screening, in which most cervical cancer cases occur in women 50 years of age and older.

## Figures and Tables

**Table 1:** Prevalence of infection with high-risk HPV in primary screening and histological CIN2+ and CIN3 lesions in HPV-positive women in relation to age in women performing self-sampling of vaginal fluid at home

	**HPV test**				**Histology**			
**Age group (years)**	**HPV, positive, *n***	**HPV, positive, %**	**Women, *n***	**CIN2+, *n***	**CIN2+, %**	**CIN3, *n***	**CIN3, %**	
30–39	63	11.5	546	21	33	11	17
40–49	59	6.6	896	14	24	6	10
50–65	66	4.7	1408	9	14	2	3
Total	188	6.6	2850	44	23	19	10

Abbreviations: CIN=cervical intraepithelial neoplasia; HPV=high-grade squamous intraepithelial lesion.

**Table 2:** Prevalence of infection with high-risk HPV in two consecutive tests and of histological CIN2+ and CIN3 lesions in women in different age categories

	**HPV test**				**Histology**			
**Age group (years)**	**HPV, positive, *n***	**HPV, positive, %**	**Women, *n***	**CIN2+, *n***	**CIN2+, %**	**CIN3, *n***	**CIN3, %**	
30–39	35	67	52	17	49	9	26
40–49	28	64	44	13	46	6	21
50–65	25	47	53	6	24	3	12
Total	88	59	149	36	41	18	20

Abbreviations: CIN=cervical intraepithelial neoplasia; HPV=high-grade squamous intraepithelial lesion.

**Table 3:** Specificity (%) for identification of histological CIN2+ lesions in relation to age in women with a high-risk HPV infection in primary screening and in women with two short-time consecutive positive HPV tests

**Age group (years)**	**Primary test positive**	**Repeat positive**
30–39	92	96.6
40–49	94.9	98.2
50–65	95.6	98.4
Mean value	94.2	97.8

Abbreviations: CIN=cervical intraepithelial neoplasia; HPV=high-grade squamous intraepithelial lesion.
